# Investigating the Comprehension of Negated Sentences Employing World Knowledge: An Event-Related Potential Study

**DOI:** 10.3389/fpsyg.2019.02184

**Published:** 2019-10-17

**Authors:** Viviana Haase, Maria Spychalska, Markus Werning

**Affiliations:** ^1^Institute of Philosophy II, Ruhr University Bochum, Bochum, Germany; ^2^Department of German Language and Literature I, University of Cologne, Cologne, Germany

**Keywords:** negation, event-related potentials, N400, world knowledge, language comprehension, alternatives

## Abstract

Previous event-related potential (ERP) studies comparing affirmative and negative sentences revealed an N400 for semantically mismatching final words, resulting in a larger N400 for false relative to true affirmative sentences and an opposite effect for negative sentences. Hence, the N400 was independent of the presence of a negation. However, the true negative as well as the false affirmative condition often contained entities or features from different semantic categories and thereby with weak feature overlap, such as e.g., *A cat is (not) a saw* or *Fears are (not) round*, which were then compared to true affirmative and false negative sentences containing entities with stronger feature overlap and partially even hyponomy relations, e.g., *A cat is (not) an animal* or *Planets are (not) round*. Employing world-knowledge variations, in the current study, we investigate whether increasing the feature overlap between the entities of all conditions leads to similar ERP-patterns as in the previous studies. For this purpose, we use sentences of the following type: *George Clooney is (not) an actor* vs. *George Clooney is (not) a singer* where both target words describe a similar profession and thereby function as alternatives to each other. However, in line with the previous studies, we find a truth by polarity interaction, namely, the N400 ERPs are significantly larger for false compared to true affirmative sentences, whereas the effect for negative sentences shows a reversed, though not significant, trend. Overall, the ERP-data suggest that the integration of a negation with the information in its scope is neither fully incremental nor fully delayed, which might be linked to the use of cohyponyms and to the increased feature overlap between alternatives (e.g., *actor, singer*). Additionally, questionnaire-based rating data show that affirmative sentences are perceived as more natural than negative sentences, and, moreover, that true sentences are perceived as more natural than false sentences, independent of their polarity.

## 1. Introduction

Negation is a feature of every human language and an essential element of everyday communication. The addition of a negation operator in a sentence results in a substantial modification of the sentence meaning through a reversal of its truth-value. Despite its frequent use in natural language, the presence of a negative marker seems to elicit additional processing resources during sentence comprehension, resulting in increased reading and reaction times, decreased response accuracy and differential event-related potential (ERP) responses when compared to affirmative sentences (Clark and Chase, 1972; Carpenter and Just, 1975; Fischler et al., 1983; Hasson and Glucksberg, 2006; Kaup et al., 2007; Luedtke et al., 2008; Dale and Duran, 2011; Wiswede et al., 2013; Dudschig et al., 2019). As a consequence, at least when presented in isolation, negative sentences have been argued to constitute an exception to fully incremental language comprehension (Carpenter and Just, 1975; Fischler et al., 1983; Kaup et al., 2006). Incremental comprehension refers to the real-time use of the information in the linguistic input as well as to anticipatory mechanisms regarding upcoming input. Hence, under the assumption of incremental comprehension, the negative marker would have to be integrated in real-time, without delays. While there are circumstances under which negative sentences seem to be processed incrementally—that is if the negation is pragmatically licensed—(Nieuwland and Kuperberg, 2008; Tian et al., 2010; Tian and Breheny, 2015), context-free occurrences of negation are still an open issue with regard to incrementality, and therefore, the general comprehension process of sentences containing a negation operator is still not well-understood. Employing world-knowledge variations, in the current study, we investigate how the use of cohyponyms of a joint hyperonym and thereby a higher overlap of semantic features between the negated entity and its correct alternatives modifies the processing as typically indicated by the N400 ERP, potentially leading to an incremental comprehension process. Additionally, we discuss the role of alternatives in negated sentences. Using context-free sentences furthermore allows a direct comparison to earlier studies employing similar designs.

The N400 component is a negative deflection in the event-related potential that is typically centro-parietally distributed, with a peak around 400 ms after the onset of a stimulus. It is elicited by every content word of a sentence (Kutas and Federmeier, 2000), and its amplitude has repeatedly been shown to be inversely correlated with a word's cloze probability (Kutas and Hillyard, 1984; Gunter et al., 1997; Dambacher et al., 2006; Wlotko and Federmeier, 2012), that is, with the proportion of individuals completing a specific context with that particular word (Federmeier and Kutas, 1999). It was discovered by Kutas and Hillyard (1980) in response to violations of meaning-related expectancy (e.g., *He spread the warm bread with socks*) and has since then been reported in numerous studies (Lau et al., 2009; Kutas and Federmeier, 2011). The size of the N400 has been reported to be modulated by a range of factors such as word frequency (van Petten and Kutas, 1990), atypical thematic role assignments (Weckerly and Kutas, 1999) and plausibility given world knowledge (Van Berkum et al., 1999; Kutas and Federmeier, 2000; Nieuwland and Van Berkum, 2006). More generally, the amplitude of the N400 has been shown to positively correlate with surprisal, that is, the negative logarithm of the conditional probability of the target word given the preceding context (Frank et al., 2015; Kuperberg and Jaeger, 2016). Moreover, Cosentino et al. (2017) and Werning et al. (2019) have shown that it is not only the semantic similarity between the target word and the preceding context (frequency and thematic role assignments held constant) what determines suprisal, but also the relevance of the preceding context for the target word. Furthermore, false compared to true affirmative sentences have repeatedly been shown to lead to an elevated N400 component (Fischler et al., 1983; Hagoort et al., 2004; Nieuwland and Kuperberg, 2008; Metzner et al., 2015; Dudschig et al., 2016, 2019; Spychalska et al., 2016, 2019). For negated sentences, a reversed ERP-pattern has been observed with true negative sentences, such as for example *A rose is not an insect*, eliciting larger N400 components than false negative sentences, such as for example *A rose is not a flower* (Fischler et al., 1983). This interaction of truth-value and sentence polarity also finds support in various behavioral studies (e.g., Clark and Chase, 1972; Hasson and Glucksberg, 2006; Dale and Duran, 2011) as well as in further ERP-studies (Luedtke et al., 2008; Wiswede et al., 2013; Dudschig et al., 2019). As a consequence, it has been assumed that the integration of the negative marker with the information in its scope is not executed in an incremental manner, but instead is a time-consuming process leading to negated sentences requiring additional time to be processed (Carpenter and Just, 1975; Kaup et al., 2006, 2007; Luedtke et al., 2008). It has been argued that comprehending negated sentences requires the initial representation of the underlying affirmative alternative, followed by its integration with the negation and an adapted representation (Carpenter and Just, 1975; Kaup et al., 2006), leading to additional processing time due to the two steps required. For example, to achieve a full understanding of *Barack Obama was not the president of the United States* we need to understand the semantically opposed alternative *Barack Obama was the president of the United States*. In the following, we will provide a brief summary of event-related potential studies employing world knowledge that compare the comprehension of true and false sentences of either affirmative or negative polarity.

In a combined ERP and fMRI study, Hagoort et al. (2004) investigated the integration of different types of knowledge during the comprehension of affirmative sentences (see also Metzner et al., 2015; Dudschig et al., 2016 for recent replications). While false^1^ (e.g., *Dutch trains are white and very crowded*) compared to true (e.g., *Dutch trains are yellow and very crowded*) sentences resulted in an N400 effect, semantically incongruent (e.g., *Dutch trains are sour and very crowded*) sentences elicited the highest N400. Furthermore, both false and incongruent sentences led to increased activation of the left inferior frontal cortex. Based on Hagoort et al. (2004)'s findings, the detection of a sentence's falsity and of its semantic anomaly required the same amount of time and activated the same resources. Note, however, that they led to different frequency band activations.

A larger N400 for false (e.g., *A bee is a truck*) compared to true affirmative sentences (e.g., *A bee is an insect*) was also reported by Fischler et al. (1983), who tested affirmative and negative sentences in a truth-value judgment task. Yet, additionally, Fischler et al. (1983) reported a larger N400 for true negative (e.g., *A bee is not a truck*) compared to false negative (e.g., *A bee is not an insect*) sentences, hence, the N400 amplitude was independent of the presence of the negative marker as the effect was higher for those sentences where the second noun was semantically unrelated to the first noun and therefore had low feature overlap. Based on these findings, it is difficult to disentangle the effect of truth on the N400 compared to the effect of mere semantic incongruence.

Wiswede et al. (2013) tested whether sentence-related factual world knowledge, e.g., *Yellow is not a number*^2^ or *Stones are not soft* is automatically activated as part of the comprehension process and whether it is used to evaluate the truth of affirmative and negative sentences. The participants were split into two groups. Each group had to complete two tasks. Participants of both groups had to respond to a probe task, which consisted of the words “true” and “false” appearing on the screen after 50% of the trials. Participants were asked to press one of two preassigned buttons for each of the two words, respectively. No truth-evaluation was required for this task. The second task varied between groups and occurred after the other 50% of trials. An evaluation group had to respond to a truth-value judgment task, while a control group had to indicate whether a probe sentence was identical to the stimulus sentence or not. In the analysis, ERPs time-locked to the onset of the final word of each sentence from both groups were included, independent of the task. *Group* was added as a separate factor. Wiswede et al. (2013) reported an interaction of truth-value and sentence polarity, that is, a larger N400 for conditions containing a semantic mismatch between subject and object of a sentence (i.e., false affirmative and true negative). This effect occurred in both groups but was stronger in the group who had to complete a truth-value judgment task than in the control group. They interpreted this effect as an indication that the analysis of word meaning and of semantic relations between words within a sentence occurs automatically, independent of the task. Furthermore, they reported significantly stronger N400 amplitudes for negative compared to affirmative sentences for both groups, independently of the sentence truth-value. Additionally, they observed a late negativity for false compared to true sentences in the truth evaluation group. This negativity occurred in a time window between 500 and 800 ms for affirmative sentences, but only later, between 800 and 1,000 ms, for negative sentences. Wiswede et al. (2013) concluded that truth validation is not fully automatic but goal dependent. However, this conclusion was based on a null result in the control group.

Nieuwland and Kuperberg (2008) addressed the interplay between pragmatic context and negation. Participants were presented with affirmative or negative sentences that were either true or false with respect to world knowledge and that were either embedded in a pragmatic context (pragmatically licensed, e.g., *With proper equipment, scuba diving is/isn't very safe/dangerous and often good fun*), or were presented without pragmatic context (pragmatically unlicensed, e.g., *Bulletproof vests are/aren't very safe/dangerous and used worldwide for security*). For the pragmatically unlicensed conditions, the authors observe a larger N400 for false affirmatives, false negatives and true negatives compared to true affirmatives. Hence, they did not observe an effect of truth-value in pragmatically unlicensed sentences on the N400 neither, which matches results from previous studies. For the pragmatically licensed conditions, however, they observed a higher N400 for false affirmative and false negative compared to true affirmative and true negative sentences. These results suggest that negation is implemented into the sentence-level meaning in an incremental manner at least if the negation is pragmatically licensed.

In a very recent study, Dudschig et al. (2019) investigated whether additional time to process the negation operator facilitates its integration into the sentence-level meaning. They compared correct (i.e., true and congruent) sentences to sentences containing either an incongruence or a world-knowledge violation, thus, their design resembled the one by Hagoort et al. (2004). In addition, they tested sentences containing a negation as well. In the first experiment, the negative adverb *nicht* (“not”) was placed within the sentence (e.g., *Zebras/Ladybirds/Thoughts are (not) stripy*). In the second experiment, an external negation that takes scope over the whole sentence was tested (e.g., *It is (not) true that zebras/ladybirds/thoughts are stripy*). The idea behind prepending the negation was to give the reader more time to process and integrate it with the information in its scope. The authors reported an N400 for the two violation conditions (incongruence and world knowledge) compared to the correct condition, both for affirmative and negative sentences in both experiments, that is, independent of the position of the negation operator. Therefore, prepending the negation operator to the beginning of the sentence did not facilitate an incremental interpretation^3^. Taken together, the results presented above suggest that the comprehension process of negated sentences is not fully incremental, not even if the system is given additional time to integrate the prepended negation with the information within its scope. As soon as a negation is pragmatically licensed, however, the comprehension process seems to function fully incrementally (Nieuwland and Kuperberg, 2008, see also Tian and Breheny, 2015).

The difficulty in achieving an overall interpretation of earlier studies is the rather strong variation of feature overlap and semantic category mismatch across conditions. For example, Fischler et al. (1983) made use of hyponomy relations such as for example in *A hammer is (not) a tool* which were compared to sentences as for example *A hammer is (not) a fish*, resulting in a mix of semantic categories as well as in a comparison of animated and not animated entity sets. Adding a negation to these sentences results in a true but pragmatically odd sentence compared to a false but in some contexts presumably acceptable sentence. The stimuli of Wiswede et al. (2013) show similar problems of semantic category mismatch and animacy violations. For example, they compared sentences like e.g., *Socrates is (not) a country* or *Iron can (not) fly* to sentences like *Five is (not) a number* or *Elephants are (not) small*. Additionally, those sentences that had a noun phrase as target word were preceded by the indefinite article which was, due to the word-by-word presentation, presented in isolation. De Long et al. (2005) reported that readers were able to predict specific words based on the prior occurrence of the indefinite article and the distinction between *a* or *an*, which was either followed by a word beginning with a vowel or a consonant in English (De Long et al., 2005, however, see Ito et al., 2016, 2017 for a debate regarding the replicability of these results). In German, due to grammatical gender, a similar differentiation is possible between *(k)ein* (neutral), *(k)einen* (male) and *(k)eine* (female). Accordingly, the use of stimuli in Wiswede et al. (2013) might have narrowed down the number of potential alternatives, thereby facilitating the anticipation of upcoming words in some trials, leading to heterogeneous material. In the current study that focuses on the comprehension of negated compared to affirmative sentences using world-knowledge, we avoid mixing semantic categories as well as animacy violations. Instead, the current study uses a true description of a publicly well-known person for the true affirmative condition, e.g., *George Clooney is an actor* which is then compared to a false version, e.g., *George Clooney is a singer*^4^. Importantly, the false version was created by using a different profession of public life denoted in a noun as well, thereby increasing the overlap of semantic features compared to the respective true sentence. Additionally we aimed at increasing this overlap by avoiding combinations of professions from rather unrelated fields, e.g., religion and sports. For the negative sentences, the adverbial negative marker *not* is added to these sentences, resulting in a false, e.g., *George Clooney is not an actor*, and a true sentence, e.g., *George Clooney is not a singer*. Using cohyponyms of the hyperonym “profession” across all conditions and increasing the feature overlap between the critical words across conditions, e.g., *actor, singer*, we aim at maximizing the coherence of all sentences to investigate how it affects the comprehension process of negated sentences.

In everyday conversation, negation does not only create a semantic opposition, but furthermore, it licenses the truth of alternatives. In isolated negated sentences, anticipating upcoming content is relatively difficult since the set of true sentence continuations for a negative sentence is vast compared to the relatively small set of true sentence continuations for an affirmative sentence. Logically, every member of the set of *not p* is a potential alternative to *p*. However, during the fast and efficient process of language comprehension, anticipating all potential alternatives would be costly for the cognitive system and furthermore would be highly inefficient since it requires maintaining an infinite amount of alternatives. In principle, potential alternatives can be found along various dimensions, depending on the type of verb that is used and depending on the scope of the negation. For example, in a sentence like *Rachel did not bake the bread*, potential alternatives for the negation can be found along the dimension of the actor, along the dimension of activities and along the dimension of the patient, that is, Rachel could have baked something else, e.g., a cake, she could have done something else to the bread, e.g., cut it, or someone else could have baked the bread^5^. As the example demonstrates, alternatives are semantically related to the negated information (e.g., entity, event). Here, we focus on the dimension of professions that are denoted as nouns in our design. In the above example, reading a sentence fragment like *George Clooney is…*, the reader may anticipate potential content related to this specific person, his profession, career, success, resulting in an expectation of words like e.g., *actor, successful, rich, famous,…*. Instead, for the respective negated sentence *George Clooney is not…*, in theory, every alternative that would make the affirmative sentence false could be anticipated. As a result, the reader might find herself in a situation of not being able to anticipate anything if presented with such a sentence in isolation. However, not all content is equally likely to occur, that is, some potential alternatives are more likely to occur than others. Due to the contextual invariance of negation (Mohammad et al., 2013; Kruszewski et al., 2016), that is, negations typically occurring in the same contexts as their affirmative counterparts, a certain feature overlap between the true continuations for an affirmative sentence and true continuations for its negated counterpart can be assumed. Accordingly, cohyponyms are straightforward alternative candidates. However, some cohyponyms, e.g., professions in the above example, seem more suitable for the negative sentences, than others. Categorization research suggests that many human categories are taxonomic, that is, items are grouped together on the basis of shared perceptual and functional features (Kay, 1971; Rosch et al., 1976). Membership within a category is gradual, determined by whether and how many features an item shares with other members of a category (e.g., Rosch, 1973, 1975). Assuming that we anticipate potential alternatives during online sentence comprehension, a gradual spread of activation in a semantic network can be assumed, in which the level of activation depends on the overlap of features. For example, other related professions as e.g., *a singer, a stage director, a producer* intuitively seem more plausible as a continuation of *George Clooney is not* than less related professions, e.g., *an architect, a pharaoh, an astronomer* would be, and certainly seem more plausible than true “out of category”-alternatives, e.g., *a bread, a dog, a hammer* that are not cohyponyms.

Our sentences are all of the form *X war einmal/nicht Y in Z* (X was once/not Y in Z) or *X ist derzeit/nicht Y in Z* (X is currently/not Y in Z) where *X* denotes a publicly well-known person, *Y* is a noun referring to a profession, and thus, is a cohyponym of the hyperonym “professions” and is the target word in this experiment, and *Z* refers to a location which can be either a country, a city or a region. The sentences in our experiment all have an SVO-structure with V being the simple past or simple present of the verb *sein* (“to be”). In the negative sentences, the negative adverb *nicht* (“not”) was placed between the verb and the object, which is the unmarked position for the negative marker in German. To keep sentence length equal between conditions, the adverbs *einmal* (“once”) or *derzeit* (“currently”) were inserted into the affirmative sentence, depending on its tense (see Dudschig et al., 2019 for a similar procedure). Hence, the two factors are *Polarity* (affirmative, negative) and *truth-value* (true, false) resulting in a 2 × 2 design. The final prepositional phrase did not alter the truth-value of the sentences and was added to avoid an overlap of effects elicited by the manipulation in the design that could be overlapping with a potential sentence final wrap-up effect. Wrap-up effects in reading are assumed to reflect increased processing associated with intra- and inter-clause integration (Just and Carpenter, 1980; Rayner et al., 2000; Hirotani et al., 2006; Warren et al., 2009). An example of the four conditions is given in Table 1. Each sentence was followed by a probe word for which participants had to decide whether it was contained in the previous sentence or not. Employing a probe verification task instead of a truth-value judgment task allows to avoid a potential confound of effects resulting from mere sentence comprehension with effects elicited by the engagement in explicit truth-value judgment. At the same time, the task is more natural than explicit truth-value judgment and requires participants to pay attention to the sentences. Additionally, the type of world knowledge violations we use might sometimes be difficult to be evaluated with a 100% certainty. For example, *George Clooney is not a singer* might seem intuitively correct in terms of world knowledge. However, strictly speaking, to be able to evaluate the truth of this sentence, we would have to have more knowledge about this person to assess whether, e.g., in private, he likes to sing. Such knowledge, however, is not relevant for the current task and is not at the focus of this experiment.

**Table 1:** Example of the experimental conditions in German with English translation.

	**True**	**False**
Affirmative	George Clooney ist derzeit Schauspieler in den USA.	George Clooney ist derzeit Sänger in den USA.
	George Clooney currently is an actor in the USA.	George Clooney currently is a singer in the USA.
Negative	George Clooney ist nicht Sänger in den USA.	George Clooney ist nicht Schauspieler in den USA.
	George Clooney is not a singer in the USA.	George Clooney is not an actor in the USA.

While the overall design of our study resembles the design of the experiments by Dudschig et al. (2019), Fischler et al. (1983), and Wiswede et al. (2013), there are various differences between them. As described above, in contrast to other studies, but especially in contrast to Fischler et al. (1983), across all conditions we use cohyponyms of the hyperonym “profession” thereby avoiding a mix of semantic categories and increasing the overlap of features between entities across conditions. Hence, we use animate entities only. In the experiment by Wiswede et al. (2013) the target word consisted either of an adjective, a noun or a noun phrase and it was preceded by either adverbial negation *nicht* (“not”) or quantifier negation *kein* (“no”) which have different scope. Instead, our target word is always a noun and the negative marker does not vary. In contrast to Dudschig et al. (2019), in our study, it is the critical word itself that alters between conditions, whereas in their study, it was the first noun of a sentence that differed while the critical word was identical in all conditions.

Semantic knowledge, world knowledge and language comprehension in general are subject to individual differences. For example, a number of studies reported an absence of predictive processes under certain circumstances, e.g., in children with low vocabulary scores (Borovsky et al., 2012), in older persons (Federmeier et al., 2002; Federmeier and Kutas, 2005; DeLong et al., 2012; Wlotko et al., 2012), in second language learners (Martin et al., 2013) and schizophrenic patients (Kuperberg, 2010). While such findings may suggest that certain speaker groups do not engage in predictive processing, it might be possible as well that these speakers anticipate upcoming input during comprehension, but that some of the computations involved are still incomplete when the relevant input arises (Chow et al., 2018). An incomplete computation of the negated sentence meaning that is only completed later in time is consistent with previous studies by Kaup et al. (2006) and Luedtke et al. (2008), as well as Dudschig et al. (2019). Here, we were interested in a potential correlation of working memory capacities and the seemingly time-consuming comprehension process for negated sentences. The high variability of the results of individual subjects reported in Fischler et al. (1983) further motivates controlling for a correlation of individual factors on ERP-results.

Despite the use of cohyponyms and thereby the increased feature overlap of the target words between conditions of our design, we still expect a larger N400 for false compared to true affirmative sentences, in line with earlier experiments (Fischler et al., 1983; Hagoort et al., 2004; Wiswede et al., 2013; Metzner et al., 2015; Dudschig et al., 2016, 2019). This comparison functions as a control comparison, that is, a complete absence of this effect might suggest that the high feature overlap resulted in a similarity between the true and false alternatives that was too strong to be noticed immediately. Furthermore, we hypothesize the modulation of feature overlap between the negated noun and its alternatives to facilitate an incremental comprehension process by increasing the chances to anticipate upcoming content in the negated sentences as well. Here, we use the term *anticipation* to refer to a potential pre-activation of upcoming content in the linguistic input as a result of overlapping features with previously encountered material already processed. The present study neither aims at investigating the automaticity of this process, nor at explicitly tackling the question of whether the N400 reflects expectancy, prediction or integration. As mentioned earlier, in a semantic network that is organized in taxonomies, a gradual spread of activation can be assumed, depending on the overlap of features between words. Due to the increase of overlapping features between the negated noun and potential alternatives we expect a facilitation of the comprehension process. Therefore, we hypothesize a smaller gap between the reaction times and response accuracies as well as reduced N400 effects. Furthermore, the inversion of the N400 for negated sentences, with true negated sentences eliciting higher N400s than false negated sentences (Fischler et al., 1983; Wiswede et al., 2013; Dudschig et al., 2019) might be changed, resulting in a larger N400 for false compared to true negative sentences. If the modulation of feature overlap between true and false alternatives does not affect the processing, we expect our results to match earlier studies (Fischler et al., 1983; Wiswede et al., 2013; Dudschig et al., 2019), hence, then we expect a larger N400 for false compared to true affirmative sentences, and a larger N400 for true compared to false negative sentences. Additionally, we hypothesize a correlation with working memory capacities, resulting in lower N400 effects for people with low working memory capacities. Engaging in anticipatory mechanisms can be expected to be more difficult with comparatively low capacities to store this information, potentially leading to a reduction or even absence of such mechanisms. Instead, high working memory capacities may enable the pre-activation of a range of alternatives, resulting in stronger N400s for each of them.

## 2. Method

### 2.1. Participants

Thirty-six (fifteen male) students of local universities participated in the experiment (age: 18–38, mean: 26.44, *SD*: 4.31) and were reimbursed for their participation or received course credit. All participants were right-handed monolingual German native speakers who were born in Germany and grew up there. The latter selection criterion was applied to increase the likelihood that they are familiar with the names used in the stimuli sentences. Part of the names are nationally well-known, e.g., due to activities on TV in Germany or in German politics, but not necessarily internationally well-known. They all had normal or corrected-to-normal vision and no history of psychological or neurological problems.

### 2.2. Material

We created 40 pairs of professions (e.g., *actor, singer*). Out of these 40 pairs we created 40 stimuli sets consisting of two true (at the time of data collection) and two false sentences by adding a negation into two of them, hence, the stimuli material consisted of 160 sentences. To avoid repetition effects and direct contradictions within the material, we split the sentences into two lists. Each participant saw only one of the lists. To do so, each quadruple of sentences was assigned two celebrities that matched the true affirmative version equally, thus, a total number of 80 celebrity names was used within the stimuli sentences. In one list, the true affirmative and the true negative of a set were assigned one person (e.g., *George Clooney*), while the false affirmative and the false negative were assigned another person (e.g., *Angelina Jolie*)^6^. With this division, we avoided the contradiction between true affirmative (e.g., *Angelina Jolie is an actor*) and false negative sentences (e.g., *Angelina Jolie is not an actor*) within one list (see e.g., Yurchenko et al., 2013 for a similar procedure). Hence, each critical word appeared four times within a list, once per condition and twice with the same name. Target words had a mean frequency of 11.49 (*SD* = 2.55, range 7–18)^7^. Within one quadruple, the mean difference in frequency was 2.55, *SD* = 1.72. When creating the stimuli, we checked LSA-values (Landauer et al., 1998) of the English translation of the two profession-hyponyms (e.g., *actor/singer*) of each set of conditions^8^. Since both were combined with the same person, the information obtained helped to approximate conceptually related professions and to combine them accordingly. Across conditions, our LSA-values are within the range −0.03 to 0.37 (with one outlier at 0.67), mean = 0.14, *SD* = 1.40.

The material has been created with the help of a questionnaire which was completed prior to the experiment. The online questionnaire consisted of two different parts and was completed by 45 German speakers (21m, age range 19–34 years, mean: 24.46, SD: 4.99) who were born in Germany and grew up there. None of them participated in the EEG experiment. In the first task, participants had to rate how well they know^9^ the person whose name was shown to them one by one. They were asked to indicate their response on a four point Likert scale and they were informed in the following way about the scale: 4 = you know a person's name and profession; 3 = you know to whom the name refers to but have little knowledge about that person, e.g., you roughly know that somebody is from politics; 2 = you hardly know a person, that is, you heard the name before but do not know who that person is; 1 = you do not know a person at all. An example was used to demonstrate the distribution of the scale. The questionnaire contained a selection of 113 female and male publicly well-known persons, covering the categories film, sciences, humanities, arts, music and politics in both past and present. For the material of the ERP-study, only those names that got a mean of 3 or higher were included in the experimental material. In total, 57 names were selected from the questionnaire [mean across all selected names = 3.63 (range 3.05−4), mean *SD* = 0.62 (range 0−0.96)]. Due to the unexpectedly high number of names that needed to be excluded due to mean values lower than 3, additionally 23 names were included that were not rated in the questionnaire^10^.

In the second part of the questionnaire the participants saw the same celebrity names in a sentence completion task. They read the beginning of a sentence of the type *X war* (X was) or *X ist* (X is), depending on whether the person is still alive and still active in their profession, where *X* is the name of a person. They were instructed to fill in a noun that they think best describes the publicly known profession of that person. The profession that is mentioned in our true affirmative sentences is the profession that the person is on average mostly known for. We took into account the answers given in the second part of the pre-test questionnaire as well as synonyms, hyponyms and hyperonyms (e.g., *artist, painter*) to these answers. When creating false sentences attention was paid to the semantic relatedness. In false affirmative sentences, the profession was taken either from the same or from a close semantic field, e.g., music and arts. We avoided to combine a person with a totally unrelated profession. For example, when creating a false sentence for a musician, a profession from the field of arts (e.g., music, painting, film) was chosen rather than a profession from politics. Furthermore, we avoided combining a person from the past with a relatively modern profession (e.g., show master). Predominantly or exclusively male professions (e.g., dictator, Pope) were not assigned to female names. The negative sentences were derived from the affirmations by adding the negative marker *nicht* (“not”).

All sentences ended with a prepositional phrase specifying the true origin of the subject of the sentence (e.g., *from Spain, in Rome*). The verb of the sentence was either the simple present or the simple past of *sein* (“to be”), depending on whether the person is still alive and still active in that field. To keep the sentence length stable and to make affirmative and negative sentences fully comparable, an adverb was inserted in the affirmative sentences after the verb (hence, at the position where the negation is located in the negative sentences). For the sentences using simple present the adverb *derzeit* (“currently”) was used, for those sentences using simple past the adverb *einmal* (“once”) was used. Those adverbs were chosen as fitting best as a counterpart to the negative marker and were closest in frequency^11^ compared with the negative adverb.

Additionally, a total of 76 filler sentences was included to increase the variability of the material. They all had the same structure as the stimuli sentences, but used different professions and other names, including cartoon figures as well. The adverb in the affirmative sentences was varied [*eigentlich* (“actually”), *offenbar* (“obviously”), *bekanntlich* (“as is known”), *damals* (“back then”), *heute* (“today”)]. The 76 sentences resulted from 19 quadruples each consisting of true and false affirmative and negative sentences. Hence, the overall distribution of affirmative and negative, true and false sentences was not altered by the fillers.

For the probe task a word in capital letters appeared on the screen and subjects had to decide by pressing a button whether this word was contained in the previous sentence or not. In 50% of the trials the probe word was part of the previous sentence. Words from all sentence positions were pseudo-randomly used in the probe task to avoid that participants would selectively focus on specific words due to the task. In case of incorrect probes, words of the same grammatical categories were used.

The stimuli were rated in two *post-hoc* online-questionnaires regarding their perceived naturalness and their perceived truth-value. The material was split into two lists, as described above for the experiment. The first questionnaire consisted of list A for the naturalness ranking and list B for the truth-ranking, while the second questionnaire consisted of list B for the naturalness ranking and list A for the truth-ranking. Hence, each questionnaire consisted of 320 questions, split into two sections with different rating tasks. The main purpose was again to avoid repetitions and contradictions within one list. The questionnaire was designed in Qualtrics and distributed via the platform Prolific, where participants received payment for their participation. Selection criteria regarding native language, provenience and age were kept identical to those for the ERP-study. In the first part of each questionnaire, participants were asked to rate each sentence regarding its naturalness on a 4-point Likert-scale (4 = natural, 3 = rather natural, 2 = rather unnatural, 1 = unnatural). They were informed that naturalness is not necessarily correlated with truth-value, and that they are therefore allowed to rate false sentences as natural and true sentences as unnatural, if necessary. In the second part, participants were asked to rate each sentence regarding its truth vale, again on a 4-point Likert-scale (4 = true, 3 = rather true, 2 = rather false, 1 = false). We asked them to complete the questionnaire without help. Within one part, the order of sentences was randomized. Each questionnaire was completed by 40 participants, that is, 80 participants (53 male) rated the material in total (mean age 24.68 years, *SD* = 4.58, range 18−39); participants who took part in the first questionnaire were not allowed to complete the second questionnaire.

### 2.3. Procedure

Upon arrival and after being informed about the procedure of the experiment the participants signed a consent of participation in accordance with the Declaration of Helsinki. Afterwards, participants filled in a translated version of the *Edinburgh Handedness Inventory* test (Oldfield, 1971), and a demographical questionnaire asking for age, handedness, education, vision, medication and neurological and psychological history. Subsequently, they completed two pretests which are part of the WAIS such as a computerized version of the Reading Span (van den Noort et al., 2008) and the Digit Span forward and backward. They furthermore filled in the *Autism Spectrum Quotient Questionnaire* (AQ) (Baron-Cohen et al., 2001), which is a self-assessment questionnaire that measures traits of the autistic spectrum disorder (ASD) in healthy adults with normal IQ, such as social skills, communication skills, imagination, attention to detail, and attention-switching, which have been reported to be correlated with differences in language comprehension, especially when comparing underinformative to informative sentences (see for example Nieuwland et al., 2010, but see also Spychalska et al., 2016)^12^.

The EEG-measurement was conducted in an electrically and acoustically shielded cabin. Participants were seated in front of a screen and a *Cedrus* response box with five buttons out of which the right and the left button were needed for the responses. After the preparation of the electrode cap subjects were given a written instruction and consecutively did a training session consisting of seven example trials. The experiment was programmed in Presentation. No feedback was given throughout the experiment. Participants were asked to attentively read the sentences and respond to the probe task. The experiment was divided into six blocks with breaks in between. The net measurement time was approximately 45 min.

The sentences were displayed on the screen in word-by-word manner in black color against a gray background (to avoid strong contrast, see Gunter et al., 1999). Each trial began with a fixation cross that was presented for 800 ms. The name was presented for 600 ms, followed by a 200 ms blank screen. The verb as well as the negation/adverb were each presented for 400 ms with a 400 ms blank each. The target word as well as the final phrase were each presented for 500 ms. After the target word, the blank lasted for 500 ms, after the final phrase until the occurrence of the probe word the blank lasted 1,000 ms. The probe word was presented maximally 3,000 ms. To respond to the probe verification task participants had to press a button; the probe word disappeared as soon as the participant clicked a response button. The assignment of the right and left button for true and false responses was counterbalanced across subjects. All participants remained naive regarding the purpose of the study.

To assess the participant's knowledge about the stimuli used in the experiment, after they completed the experiment, they filled out a digital questionnaire. It was designed in the same way as the pre-test (see section 2.2) and included every name used in the stimuli sentences (i.e., 80 names).

### 2.4. EEG Recording and Preprocessing

The EEG was recorded with a 64 channel ActiCap system by BrainVision, band-pass filtered at 0.01-250 Hz and sampled with a frequency of 500 Hz. AFz served as Ground, FCz as physical reference during the recording. To control the vertical and horizontal eye movements four electrodes (FT9, FT10, PO9, and PO10) were removed from their determined location and were placed over and under the right eye as well as on both temples to measure the electrooculogram (EOG). All impedances were kept below 5 kΩ. The data was processed using the Brain Vision Analyzer 2.1 software. We applied an offline band-pass filter of 0.1-30 Hz. All trials with an absolute amplitude difference higher than 200μ*V*/200*ms* or with an activity lower than 0.5μ*V* in intervals of 100 ms or longer were automatically rejected. The maximal allowed voltage step was 50μ*V*/*ms*. Eye-blinks and eye-movements were corrected by a semi-automatic independent component analysis. The data was re-referenced to the linked mastoids (TP9, TP10) and then segmented into epochs of 1,000 ms, beginning at the onset of the second noun, with a −200 ms baseline. The baseline correction serves to remove differences due to drifts, while avoiding a distortion of the post-stimulus ERPs that might result from transient differences between conditions in the baseline interval (Wolff et al., 2008). Before averaging, any segments with remaining physical artifacts lower than −90μ*V* or higher than 90μ*V* were removed. Across subjects, the minimum of preserved segments was 25 out of 40, however, for most subjects, at least 30 segments per condition (i.e., at least 70%) were preserved. Four participants had to be excluded from the ERP-data analysis due to excessive artifacts leading to a loss of more than 50% of segments per condition for three of them, and due to strong signal drifts on multiple electrodes in the fourth participant. One data set was excluded due to a technical problem during recording, hence the ERP-analysis is performed on 31 participants.

## 3. Results

### 3.1. Behavioral Responses to the Probe Task

The mean accuracies and mean response times to the probe task for 31 subjects are shown in Table 2. For the behavioral responses the non-parametric Friedman test, which, unlike ANOVA, can be used for samples that are not normally distributed, indicated that the mean accuracy to the probes differed across the four conditions: χ^2^(3) = 12.457, *p* = 0.005 (*N* = 31). Based on the Wilcoxon *post-hoc* analysis, the effect results from a lower mean accuracy for false negative compared to false affirmative sentences (z = −2.310, *p* = 0.019) and from lower mean values for false negative compared to true negative sentences (z = −2.118, *p* = 0.033).

**Table 2:** Mean accuracy in the Probe Verification Task in percentage and mean reaction times in milliseconds for all four conditions; standard deviations are indicated in brackets.

	**Accuracy**		**Reaction time**	
	**True**	**False**	**True**	**False**
Affirmative	97.99 (2.6)	97.61 (3.04)	885.01 (204.567)	924.173 (229.216)
Negative	97.35 (3.00)	96.32 (2.88)	911.737 (204.48)	896.52 (195.77)

*N = 31*.

For the reaction times the parametric repeated measures ANOVA revealed an interaction Polarity^*^Truth [F(1, 30) = 13.758, *p* = 0.001, partial η^2^ = 0.314] but no main effect for *Polarity* (*F* > 0.05, *p* > 0.5) and no main effect of *Truth* (*F* > 3, *p* > 0.08). We broke down the interaction by *Polarity*. For affirmative sentences, the ANOVA shows a main effect for *Truth* [F(1, 30) = 13.290, *p* = 0.001, partial η^2^ = 0.307], with responses to false probes on average taking longer than responses to true probes (Δ_(*False, True*)_ = 39.16 ms). There was no effect for negative sentences (*F* > 0.2, *p* > 0.1).

### 3.2. ERP-Results

The ERPs elicited by the target word of the sentence were evaluated in a repeated measures ANOVA with *Polarity* (affirmative/negative) and *Truth* (true/false) as within-subject factors. To analyze possible interactions with electrode positions, *Lateralization* (left/right) and *AP* (anterior/posterior) were involved as further factors, resulting in four regions of interest (ROI). Each ROI comprised 11 electrodes: left anterior (FP1, AF3, AF7, F1, F3, F5, F7, FC1, FC3, FC5, FT7), right anterior (FP2, AF4, AF8, F2, F4, F6, F8, FC2, FC4, FC6, FT8), left posterior (CP1, CP3, CP5, TP7, P1, P3, P5, P7, PO3, PO7, O1), and right posterior (CP2, CP4, CP6, TP8, P2, P4, P6, P8, PO4, PO8, O2). In all ANOVAs, all dependent variables were normally distributed and met the assumption of sphericity, unless otherwise indicated. The p-values of all pairwise-comparisons were Bonferroni corrected.

**Figure 1:**
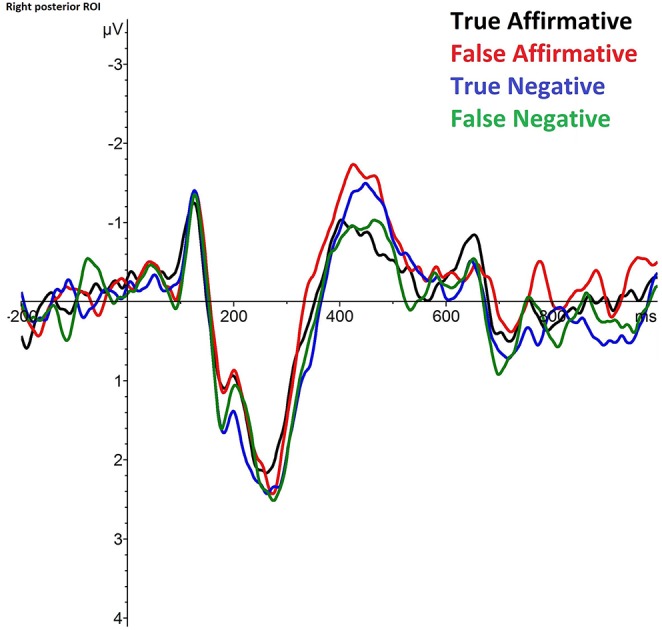
Grand average across all subjects (*N* = 31) for all four experimental conditions at the right posterior ROI, based on the average of 11 electrode positions (CP2, CP4, CP6, TP8, P2, P4, P6, P8, PO4, PO8, O2).

The visual inspection of the target word revealed an N400 component that is higher for false than for true affirmative sentences, but lower for false than for true negative sentences (see Figure 1). The ANOVA in the time-window 400–500 ms revealed an interaction AP^*^Polarity^*^Truth [F(1, 30) = 6.197, *p* = 0.019, partial η^2^ = 0.171] as well as an interaction Lateralization^*^Polarity^*^Truth [F(1, 30) = 4.540, *p* = 0.041, partial η^2^ = 0.131]. Furthermore, there is a main effect of *AP* [F(1, 30) = 21.599, *p* =< 0.001, partial η^2^ = 0.419], with the frontal electrodes on average showing more negative amplitudes than the posterior electrodes (Δ_(*Post, Front*)_ = 1.962μ*V*), as well as a main effect of *Lateralization* [F(1, 30) = 16.136, *p* =< 0.001, partial η^2^ = 0.350], with the electrodes on the right hemisphere showing more negative amplitudes than the left hemisphere (Δ_(*Right, Left*)_ = −0.863μ*V*). Subsequently, we performed ANOVAs for each region separately to break down the two interactions.

**Figure 2:**
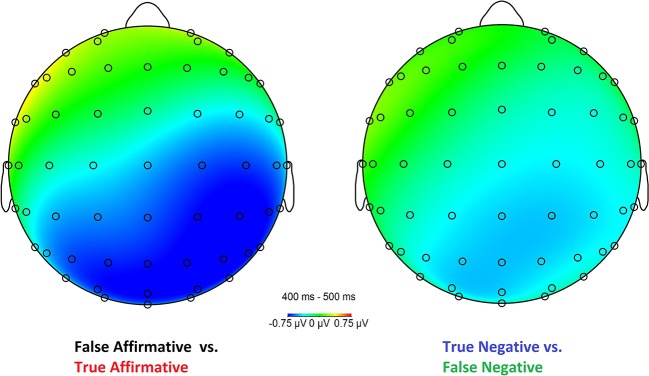
Topographical maps of the differences between false and true affirmative sentences **(Left)** and between true and false negative sentences **(Right)** in the time window 400–500 ms. *N* = 31.

The separate ANOVA for the right posterior region revealed an interaction Polarity^*^Truth [F(1, 30) = 6.616, *p* = 0.015, partial η^2^ = 0.181], with false affirmative sentences having more negative amplitudes than true affirmative sentences (Δ_(*False, True*)_ = −0.686μ*V*), and true negative sentences showing more negative amplitudes than false negative sentences (Δ_(*True, False*)_ = −0.386μ*V*). Broken down by *Polarity*, the separate ANOVAs revealed a main affect of *Truth* for the affirmative sentences [F(1, 30) = 7.453, *p* = 0.01, partial η^2^ = 0.199], but not for the negative sentences [F(1, 30) = 1.979, *p* = 0.170, partial η^2^ = 0.062]. See Figure 2 for the topographical distribution. There is no main effect of *Polarity* (*F* > 0.1, *p* > 0.7) and no main effect of *Truth* (*F* > 0.8, *p* > 0.3).

No effects were found in the remaining three regions (*F* > 0.03, *p* > 0.4). Adding *Working Memory* or *AQ-score* as between-subject factors based on Median Split brought no significant effect for that factor^13^.

The midline electrodes were analyzed separately with the factors *Polarity, Truth* and *Midline ROI* (anterior, posterior, left, right). The following electrodes are included: anterior (Fz, FCz, Cz), posterior (CPz, Pz, POz, Oz), left (C2, C3, C5, T7), right (C2, C4, C6, T8). The ANOVA revealed an interaction Polarity^*^Truth^*^Midline ROI [F(3, 28) = 2.884, *p* = 0.04, partial η^2^ = 0.088], as well as a main effect for *Midline ROI* [*F*(3, 28) = 15.348, *p* =< 0.001, partial η^2^ = 0.338]. Subsequently, we performed ANOVAs for each midline region separately to break down the interaction.

The posterior midline ROI shows a marginally significant interaction Polarity^*^Truth [F(1, 30) = 3.811, *p* = 0.06, partial η^2^ = 0.113]. The right midline ROI shows a marginally significant interaction Polarity^*^Truth [F(1, 30) = 3.508, *p* = 0.07, partial η^2^ = 0.105] as well. There was no main effect in any of the four midline regions, nor any interaction in the remaining two regions (anterior and left) (*F* > 0.009, *p* > 0.4).

### 3.3. Knowledge Questionnaire

In the first task of the post-experiment questionnaire participants had to rate each name used in the stimuli of the EEG experiment on a scale from 1–4^14^ to indicate their level of knowledge. The second part of the questionnaire consisted of a sentence completion task asking them to complete *X war/ist* (X was/is), with X being the names used in the experiment. For the first task, the mean rating across all items and participants was 3.67 with SD = 0.82. Six subjects had mean values below 3, however, we did not exclude them from the analysis since their responses to the second part indicated that they had the required knowledge to assess the sentence truth-value. As indicated in the description of the material, 23 names that were not part of the pre-test were included in the stimuli. To assess our participants knowledge about these 23 items, we calculated the mean values for these 23 names separately, in addition to the analysis above. These 23 items received a mean of 3.05 with *SD* = 0.96. Among these 23 names, those items that received mean values below 3 interestingly received correct answers in all but two cases in the second task, indicating that our subjects had enough knowledge to recognize the truth of the stimuli sentences in most cases.

### 3.4. *Post-hoc* Questionnaire: Perceived Naturalness and Perceived Truth-Value Rankings

The *post-hoc* questionnaire was completed online by 80 participants who did not take part in the ERP-study. The mean ratings with standard deviations for perceived naturalness and perceived truth-values are shown in Table 3. Regarding the perceived naturalness, the ANOVA revealed a main effect of *Polarity* [F(1, 79) = 934.424, *p* < 0.001, partial η^2^ = 0.922] and a main effect of *Truth* [F(1, 79) = 21.225, *p* < 0.001, partial η^2^ = 0.212], as well as an interaction Polarity^*^Truth [F(1, 79) = 7.196, *p* = 0.009, partial η^2^ = 0.083]. Broken down by *Polarity*, the separate ANOVAs revealed a main effect of *Truth* for the affirmative sentences [F(1, 79) = 17.212, *p* < 0.001, partial η^2^ = 0.179], with lower mean ratings for false compared to true affirmative sentences (Δ_(*True, False*)_ = 0.22μ*V*). The negative sentences showed a main effect of *Truth* [F(1, 79) = 4.128, *p* = 0.046, partial η^2^ = 0.05] as well, with slightly lower mean ratings for false compared to true negative sentences (Δ_(*True, False*)_ = 0.06μ*V*).

**Table 3:** Mean values of the perceived naturalness of the stimuli (scale: 4 = natural, 3 = rather natural, 2 = rather unnatural, 1 = unnatural) and mean values of the perceived truth (4 = true, 3 = rather true, 2 = rather false, 1 = false) for all four conditions; standard deviations are indicated in brackets.

	**Naturalness**		**Truth**	
	**True**	**False**	**True**	**False**
Affirmative	3.39 (3.42)	3.17 (3.5)	3.41 (6.2)	1.68 (5.48)
Negative	2.37 (1.71)	2.31 (1.92)	3.2 (5.79)	1.58 (5.44)

*N = 80*.

Regarding the perceived truth-value, the ANOVA revealed a main effect of *Polarity* [F(1, 79) = 5.464, *p* = 0.022, partial η^2^ = 0.65] with affirmative sentences receiving higher mean ratings than negative sentences (Δ_(*True, False*)_ = 0.16), and a main effect of *Truth* [*F*(1, 79) = 876.789, *p* < 0.001, partial η^2^ = 0.917] with true sentences receiving higher mean values than false sentences (Δ_(*True, False*)_ = 1.67). There was no interaction Polarity^*^Truth (*F* > 0.593, *p* > 0.44).

## 4. Discussion

The comprehension of isolated negated sentences has been argued to be an exception to incremental language comprehension, which is based (inter alia) on evidence from a range of event-related potential studies showing an interaction of polarity and truth-value. These studies reported true negated sentences eliciting higher N400 ERPs than false negated sentences suggesting that the N400 is driven by priming relations within sentences rather than by sentence truth-value (Fischler et al., 1983; Wiswede et al., 2013; Dudschig et al., 2019). Our study examined the comprehension of negated sentences in comparison to affirmative sentences, employing world knowledge in true and false sentences.

In contrast to earlier studies that used similar designs, we make use of cohyponyms, thereby increasing the overlap of features from the set of true alternatives for the affirmative sentence and the set of true alternatives for the negative sentence. Thereby, we aimed at facilitating the anticipation in the negated sentences to investigate whether it leads to similar ERP-patterns as in previous studies (Fischler et al., 1983; Wiswede et al., 2013; Dudschig et al., 2019). *Anticipation* here is used to describe potential pre-activations of upcoming content in the linguistic input that results from a feature overlap with previously processed content. We hypothesized to find an N400 effect for false compared to true affirmative sentences, in line with earlier studies using world knowledge violations (Fischler et al., 1983; Hagoort et al., 2004; Nieuwland and Kuperberg, 2008; Wiswede et al., 2013; Metzner et al., 2015; Dudschig et al., 2016, 2019). For negative sentences, we hypothesized a reduction of the N400 effect for true vs. false sentences, or an N400 for false compared to true negative sentences.

We observe an interaction of truth-value and polarity which is driven by reversed effects for affirmative and negative sentences, that is, by larger N400 ERPs for false compared to true affirmative sentences, but smaller N400 ERPs for false compared to true negative sentences. Split by *Polarity*, the effect is significant only for affirmative sentences, with a larger N400 for false compared to true sentences. For negated sentences, there is no significant effect, but the trend goes in the same direction as in earlier studies, thus, negation reverses the N400 pattern with more negative amplitudes for true compared to false sentences. A significant interaction is observed in the right posterior region, matching the typical topography of the N400 component for written sentences (Kutas et al., 1988; Kutas and Federmeier, 2011). Overall, our amplitude differences seem to be smaller compared to earlier studies, which can be a result of the increased feature overlap between the alternatives used as critical words in our sentences. However, the decrease in amplitude size might as well be at least partially affected by the use of a probe task instead of a truth-value judgment task as in earlier studies which is in line with the results by Wiswede et al. (2013), who observed reduced amplitude differences in the N400 time window for the control group compared to the truth-evaluation group.

The ERP-results match the observations for reaction times to the probe task which show longer response times for false compared to true affirmative sentences, but no difference between negative sentences. Additionally, there is no significant difference between responses to affirmative and negative sentences, yet the means show that across conditions, responses to the false affirmative sentences were the slowest.

Furthermore, we assumed that engaging in anticipatory mechanisms can be expected to be more difficult for individuals with lower working memory capacities (WMC), which eventually may lead to an absence of such mechanisms in this group. Therefore, we hypothesized that participants with low WMC will show lower N400 effects than people with high WMC since the latter may anticipate a range of alternatives more easily, resulting in stronger N400s for each of them. While the visual inspection of the data shows that the N400 amplitudes are generally reduced for people with low working memory capacities, the correlations with the working memory tests (Reading Span and Digit Span) were not significant. The results partially match the findings from Otten and van Berkum (2009) who investigated the impact of individual WMC in an ERP-study. They report individuals with low as well as with high working memory capacities to predict specific upcoming words. Both groups show an early negative deflection for unexpected compared to expected determiners in predictive stories. Hence, the ability to rapidly and automatically predict upcoming linguistic material seems to be independent of a person's WMC, that is, of their ability to temporarily store and manipulate information. At the same time, however, in the study by Otten and van Berkum (2009), low working memory readers additionally showed a late negativity to linguistic material that was inconsistent with the participant's prediction, suggesting additional processing. Possibly, this additional neural response reflects increased demands of the adjustment or the suppression of the original prediction. While this result matches the findings of Luedtke et al. (2008), who report an enhanced negativity for words following the negative quantifier *no* (e.g., *In front of the tower there is no ghost*), it does not match the results from the current study. Since we did not select participants based on their working memory capacities and since our participants are mostly students in a certain age range, the variation of values they obtained in the different pre-tests are mainly pooled at the upper and upper-central part of the respective scales. Therefore, the variation resulting from grouping them into high-working-memory-readers and low-working-memory-readers based on a median split might have been too low to become significant, especially since the N400 amplitude differences in our study are generally reduced. We were furthermore interested to see whether the N400-ERPs for negated sentences, which are typically underinformative, at least when presented in isolation, correlate with the AQ-score of participants, similarly to Nieuwland et al. (2010). We do not find such a correlation, matching the results by Spychalska et al. (2016). Again, we did not select participants based on their scores in the AQ-test and neurological and psychological disorders were an exclusion criterion for our study.

One potential explanation for larger effects within the affirmative sentences is the anticipation of upcoming content in the true affirmative compared to the false affirmative sentences due to a higher overlap of features associated with e.g., *Angelina Jolie* and *actress* than with *singer*. In negative sentences, instead, the anticipation of alternatives is usually more difficult, unless the context provides only a binary choice of alternative options (Orenes et al., 2014). The reduction of the amplitude difference between the two negated conditions might reflect a “partially incremental” integration of the negation which was facilitated due to the feature overlap within both sentences. A fully incremental integration should have led to an N400 for false compared to true negative sentences. Instead, a total absence of incremental comprehension should have led to the same amplitude differences as for affirmative sentences. Urbach and Kutas (2010) provide a similar suggestion, namely, that the interpretation of quantifier expressions as for example *most* and *few* is neither fully incremental nor fully delayed, therefore, it is argued to be “partially incremental.” Sentences with negative quantifiers have been reported to reveal similar result patterns as sentences with propositional negation, resulting in an interaction of truth and quantifier type (Kounios and Holcomb, 1992). Related to that, Nieuwland (2016) observed smaller N400s for false compared to true sentences, independent of the type of quantifier (*few* vs. *many*), however only in sentences with high cloze values for the target word. For sentences with lower cloze values, the pattern for positive quantifiers was similar, but it was reversed for negative quantifiers, that is, in sentences where the target word had a low cloze value, the true negative sentences had higher N400 amplitudes than the false negative sentences. Even though our affirmative and negative sentences are not matched with regard to cloze value, the increased feature overlap in our study might facilitate the anticipation of upcoming content in a similar way. The smaller difference between amplitudes for our negative sentences might therefore reflect an approximation toward a typical N400 pattern, with false compared to true sentences eliciting larger N400s. Based on our results and the results by Nieuwland (2016), high cloze values then should further affect the N400 for sentences with propositional negation, leading to a similar pattern as is typically observed for affirmative sentences, that is, a larger N400 for false over true sentences.

Negation has been reported to lead to lower activation levels for negated probes (MacDonald and Just, 1989) and negated sentences (Tettamanti et al., 2008) in functional neuroimaging studies. As part of the related debate about negation playing some sort of inhibitory role on concepts, it has been discussed whether this attenuation also spreads to associated concepts or whether the negation of one concept actually enhances a spread of activation across associated alternative concepts (see e.g., Anderson et al., 2010). Given that negations tend to occur in the same contexts as their affirmative alternatives, the latter option seems to support an incremental and efficient comprehension process more than the former. Furthermore, without alternatives, negated sentences would be underinformative. MacDonald and Just (1989) did not find an inhibitory effect of negation on associated concepts, suggesting that those alternatives indeed became activated during the comprehension process, which matches our results. The use of alternatives furthermore depends on the negated dimension, hence, on the scope of the negation. Previous studies suggested mixed results about alternatives during comprehension. Tian and Breheny (2015) have shown that in sentences with clear scope and therefore clearer alternatives (e.g., *It is John who hasn't ironed his brother's shirt*), incremental comprehension is facilitated. However, reducing the alternatives alone does not facilitate comprehension in all cases. Nieuwland and Kuperberg (2008) used contrary adjectives such as e.g., *easy-difficult, rich-poor, safe-dangerous* as target words which, when negated, directly allow for an interpretation by replacing the negated adjective with its unique alternative. Yet, this alone did not facilitate comprehension, instead, only the pragmatic embedding of the sentences did. In our sentences, having wide scope, in principle everything could have been negated, including both nouns, the verb as well as the prepositional phrase. However, negating the first noun would require further emphasis, either by stressing it (in spoken language) or by using a cleft sentence, e.g., *It is not George Clooney who is an actor*. In theory, in our material it is the second noun and the prepositional phrase that can be interpreted as being negated either altogether or separately. We cannot exclude that participants interpreted the negation taking scope over the final phrase of our sentences. However, it is likely that they noticed that it is the profession (second noun) rendering some of the sentences true and others false because the final phrase always led to true sentences across all stimuli and across fillers.

Due to the addition of the final phrase, e.g., *in the USA*, that was added to avoid a potential overlap of negation-induced effects and the sentence wrap-up effect (Just and Carpenter, 1980; Rayner et al., 2000; Hirotani et al., 2006; Warren et al., 2009), one might argue that participants could have “waited” for this phrase to come for their intuitive truth-value judgment. However, first of all, no explicit truth-value judgment was required and under the notion of incremental comprehension the target word can be assumed to be integrated into sentence meaning before the occurrence of further input material. Secondly, the final phrase did not modulate the truth-value, but provided the true origin of the person mentioned in the sentences. Yet, intuitive truth-value judgment, despite not being required for the task, was required to achieve a full understanding of the sentence which might have been especially difficult for the true negative sentences. For example, *George Clooney is not a singer* might be intuitively easy to be judged as true. Yet, to fully assess its truth, we usually do not have enough knowledge about celebrities. As a result, participants might have achieved a full understanding only for the affirmative sentences which clearly show an N400 effect despite the implicit probe task, but not for the negated sentences. The second aspect of our design that might have had further impact on the differences across conditions is the adverb in the affirmative conditions. To keep the sentence length stable, the adverbs *derzeit* (“currently”) or *einmal* (“once”) were added into the affirmative sentences. They were chosen as the best fit under the additional constraint of having similar frequency values as the negative operator^15^. We cannot exclude that the use of these adverbs had some effect on the results, leading to the stronger N400 contrast for the affirmative sentences. While the adverb *derzeit* (“currently”) seems a relatively neutral counterpart to a negation for the sentences with present tense, the adverb *einmal* (“once”) might have had a pragmatic effect on the interpretation. While it can be understood along the lines of *used to be* when combined with the verb *to be*, some people might also interpret it more strictly as meaning *one time* which would make the interpretation of the false affirmative sentences more difficult because we do not have enough knowledge about the people described by the sentence, to exclude the possibility that, for example, *Beethoven once was a painter* is false because we might assume that maybe he indeed also painted and we simply do not know about it.

The typically reversed effect for negated sentences with the N400 being larger for true compared to false negative sentences has often been assumed to be driven by the true negative sentences and the semantic distance due to lower feature overlap between the entities within these sentences (e.g., *rose* and *insect* Fischler et al., 1983 or *George Clooney* and *singer* in our study) and therefore, the absence of priming when compared to the false negative sentences. Alternatively, the reversed ERP-pattern might be driven by the falsity of the false negative sentences not being detected. Incomplete or “shallow” processing in general refers to an incomplete interpretation of the information available in the linguistic input which results in an incomplete or underspecified representation (Frazier and Rayner, 1990; Ferreira et al., 2002; Sanford and Sturt, 2002, see also Baggio et al., 2012). It has been observed in various experiments that in certain scenarios where the semantic similarity between words of a sentence is high, readers do not detect incomplete or semantically anomalous information, thereby achieving a wrong interpretation of the sentence. Examples for these kind of “semantic illusions” are “How many animals of each type did Moses take on the ark?” (Erickson and Mattson, 1981) where readers did not detect that it was not Moses but Noah who took animals onto the arch, or “What is the holiday where children go door to door, dressed in costumes, giving out candy?” (Reder and Kusbit, 1991), where participants fail to detect that children do not hand out but collect candies. Potentially, in our study, participants failed to detect the falsity of sentences like *George Clooney is not an actor* or, in the study by Fischler et al. (1983) of *A bee is not an insect*, at least not fast enough for the difference to be reflected in the online-comprehension signature. Even though this is a null result, we point out that reaction time data further support this interpretation, as there was no difference between the responses to the negated sentences, but there was a difference between the affirmative sentences. This potential interpretation matches the results from the quantifiers study by Urbach and Kutas (2010) mentioned before, who tested sentences like *Most/Few farmers grow crops/worms* and reported an N400 for *worms* independent of the quantifier type. However, the effect was smaller for cases with negative quantifiers. At the same time, the offline plausibility judgment showed that both true sentences were rated more plausible suggesting that participants achieved a full understanding by the time the plausibility question appeared after the sentence. It has been shown in earlier studies, that manipulating the time window between an affirmative or negative sentence and a subsequently presented matching or mismatching picture leads to different results suggesting that the implementation of the negation into the sentence meaning is time consuming (Kaup et al., 2006; Luedtke et al., 2008). Our ERP-results then would not contradict those findings. At the same time, however, they suggest that an increase of feature overlap between the entities of a sentence seems to trigger a “partially incremental” interpretation of the negation operator.

The ratings of our *post-hoc* questionnaire, in which participants were asked to rate all sentences regarding their naturalness and their truth-values, each on a scale from 1 to 4, suggest that negative sentences are in general perceived to be less natural than affirmative sentences. This finding is not surprising as negative sentences are less frequent and more marked, and especially when presented in isolation are considered to be less informative than affirmative sentences, thereby violating Grice's Conversational Maxims (Grice, 1975). Furthermore, false sentences were perceived as slightly less natural than true sentences. Therefore, the reversed N400 for true compared to false negative sentences cannot be explained based on their perceived naturalness. If naturalness was the reason for the observed N400 in this and prior studies, true negative sentences should have received lower mean values with respect to their naturalness than false negative sentences. Regarding the perceived truth-value, participants responses are as expected, that is, true sentences received higher ratings than false sentences, independent of their polarity. Furthermore, affirmative sentences received higher ratings than negative sentences. It should be noted though that the standard deviations are relatively high for all four conditions, indicating that individual responses strongly varied. These results match the accuracy of responses to the main experiment with false negative sentences leading to more incorrect responses both compared to true negative as well as compared to false affirmative sentences. Given that this experiment involves world knowledge and given the addition of the adverb as well as the final phrase along with their pragmatic implications, the results of the *post-hoc* questionnaire are not surprising. Importantly though, as shown by the main effect of *Truth*, false sentences can be expected to be recognized as such, both for affirmative as well as for negative sentences. However, note that the questionnaire ratings are offline-ratings. Participants were asked to rely on intuitive judgments without thinking too long about each sentence. Yet, the presentation time of the sentences was not limited in time and furthermore, sentences were not split into single words. Instead, in the ERP-study, which reflects online-sentence comprehension, each word was presented in isolation and only for few hundred milliseconds. Taken together, the pattern from our ERP-study and from our *post-hoc* questionnaire ratings fully match the combination of online-ERP results and offline plausibility judgments by Urbach and Kutas (2010) presented above. Furthermore, one might argue that the truth-value ratings contradict the claim made above that the falsity of the false negated sentences is not detected. However, let us emphasize again that the option of the falsity of the negative sentence not being detected fast enough, hence, at the time the target word is presented, cannot be ruled out by the questionnaire. Hence, the offline questionnaire rating does not contradict the option of an incomplete computation of the negated sentence meaning that is only completed later in time.

In sum, our study can be taken as an indication for an increase of feature overlap between the entities within sentences leading to a decrease of amplitude differences between true and false negative sentences compared to earlier studies, however, the trend for negative sentences eliciting larger N400s than false negative sentences persists. Our results are in line with earlier studies, but additionally they suggest a “partially incremental” comprehension process, that is, the integration of the negation with the information in its scope is neither fully incremental nor fully delayed. Future experiments investigating the time-course of comprehension in negated sentences using different verb types and varying the position of the negative marker regarding the verb are necessary, for example to assess the role of alternatives along other dimensions as well as the role of alternatives in sentences with full verbs opposed to copula verbs in general. In addition, comparing affirmative and negative sentences with similar cloze values (cf. Nieuwland, 2016) could reveal further information about (isolated) negated sentences being processed incrementally or not.

## Data Availability Statement

All datasets generated for this study are included in the manuscript/Supplementary Files.

## Ethics Statement

The study was conducted in compliance with the Helsinki declaration and the ethical guidelines of the national research funding agency in Germany (German Research Foundation, DFG). The presented material did not contain any potentially harmful content and EEG is a non-invasive method. According to the guidelines of the German Research Foundation, for EEG experiments, an explicit ethics approval is only required if the study is conducted with persons below 18 or over 65 years old, or with patients. All subjects were adult volunteers (between 18 and 38 years old) and signed an informed consent of participation, which included information about protection of privacy and confidentiality, as well as the right to withdraw from participation at any point.

## Author Contributions

VH designed, programmed and conducted the experiment, prepared the stimuli, processed and analyzed the data, and wrote the manuscript. MS advised on the data processing, interpretation as well as and on the manuscript, and contributed to the data analysis. MW advised on the experiment design, the data processing, data interpretation, and provided the resources for the experiment.

### Conflict of Interest

The authors declare that the research was conducted in the absence of any commercial or financial relationships that could be construed as a potential conflict of interest.

## Footnotes

^1^*Falsity* means a world knowledge violation, both here as well as in the study by Wiswede et al. (2013) presented below. Instead, *congruence* refers to semantic features, e.g., *sour* is not a feature of trains.

^2^The experiment was done in German, the original sentences were for example *Gelb ist keine Zahl* and *Steine sind nicht weich*. Hence, negation was either marked using the negative quantifier *kein* (“no”) or the negative adverb *nicht* (“not”) which have different syntactic structures and different scope.

^3^However, providing additional time after the sentences (in a sentence-picture verification paradigm with the sentence preceding the picture) seemed to allow a successful integration of the negation (Kaup et al., 2006, 2007; Luedtke et al., 2008, see also Ferguson et al., 2008).

^4^Our experiment is done in German and does not require any article in this construction to yield a grammatical sentence. Thereby, the potentially facilitated predictability just described for the study by Wiswede et al. (2013) does not apply to our design.

^5^This example refers to a sentence presented in isolation and in written language. Context as well as prosody would mark the focus of the sentence, thereby limiting the number of alternatives.

^6^In German, professions usually are inflected for gender, therefore, the noun denoting the profession was adapted accordingly, resulting in e.g., *Angelina Jolie ist Schauspielerin*.

^7^Leipzig Wortschatz http://wortschatz.uni-leipzig.de/de

^8^Obtaining LSA-values for the two nouns within one stimulus sentence in the current design is problematic because of the use of proper names. Not all names can be found in the LSA-databases and they are at risk to lead to a distorted picture. For example, by checking the LSA-value for “George Clooney” and “actor”, all entries where he is referred to by “Mr. Clooney” or “Clooney” are ignored. These variations, however, are frequently used to avoid repetitions in texts and therefore are likely to occur in the text pool underlying the database. Furthermore, frequent proper names might be subject to ambiguities within these databases.

^9^They were informed that knowing a person here does not mean knowing them personally but rather knowing who this person is and what this person's field of publicly known profession is.

^10^See section 3 for their mean values from a post-experiment rating.

^11^Leipzig Wortschatz http://wortschatz.uni-leipzig.de/de: einmal (6), derzeit (7), nicht (2).

^12^We therefore considered it worth testing whether the way in which individuals process negated sentences, which are considered to be underinformative when presented in isolation, correlates with their AQ-score.

^13^A figure with the Grand Averages split by Working Memory (high vs. low) as well as a figure of the separate Grand Averages for each of the four ROIs can be found in the Supplementary Material.

^14^The scale was identical to the pre-experiment questionnaire desribed in the section 2.2 (i.e., a four point Likert scale). Participants were informed in the following way about the scale: 4 = you know a person's name and profession; 3 = you know to whom the name refers to but have little knowledge about that person, e.g., you roughly know that somebody is from politics; 2 = you hardly know a person, that is, you heard the name before but do not know who that person is; 1 = you do not know a person at all. An example was used to demonstrate the distribution of the scale.

^15^Nevertheless, we would like to point out that negative marker “nicht” is a very high frequent word (ranked 17th of all words in the database) which inevitably leads to rather strong frequency differences when compared to any adjective both in the current as well as in previous studies comparing affirmative and negative sentences.
