# Supplementary figures and images for: Investigating the Comprehension of Negated Sentences Employing World Knowledge: An Event-Related Potential Study
Source: Front Psychol. 2019 Oct 17;10:2184. doi: 10.3389/fpsyg.2019.02184 (PMC6843029; doi:10.3389/fpsyg.2019.02184)

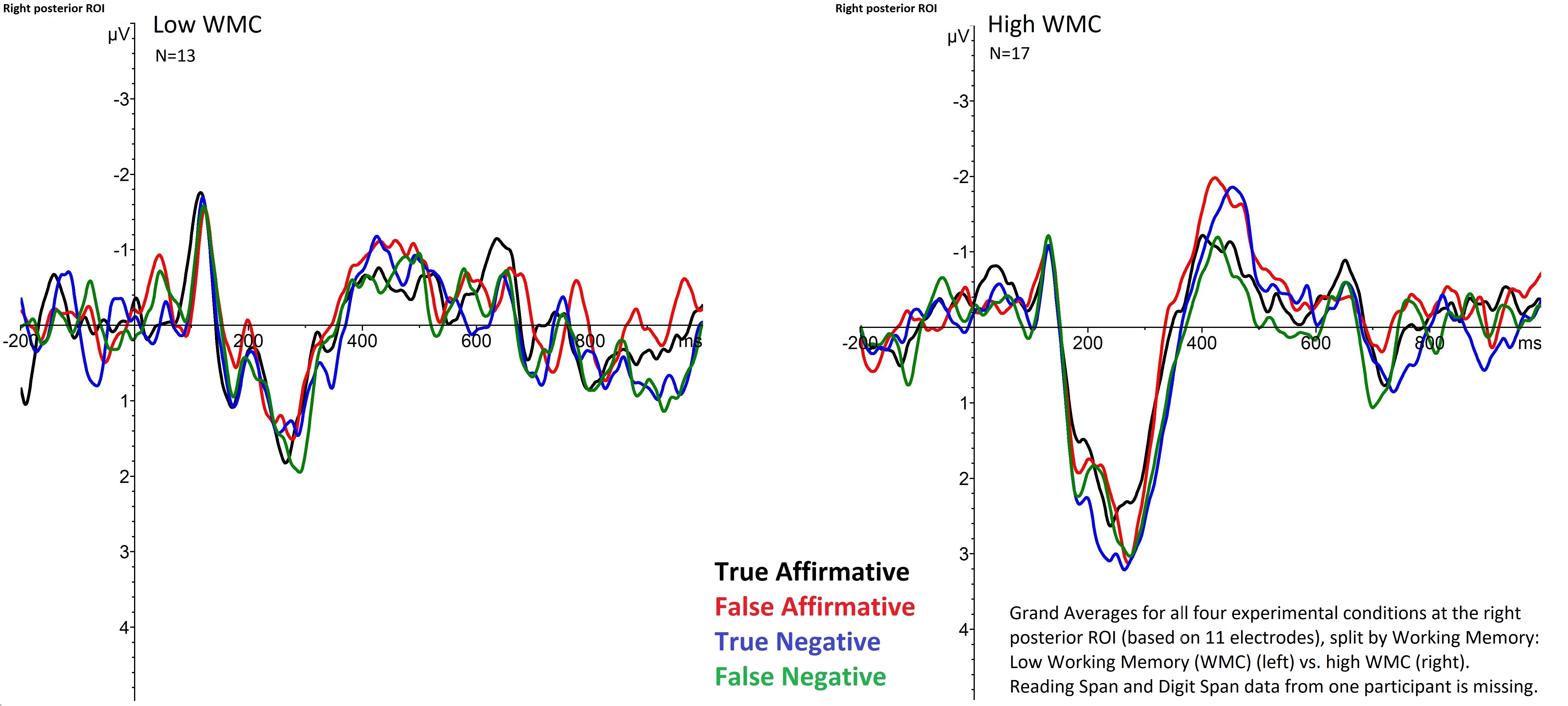

Supplement: Supplementary file 2 [file Image_3.jpg]

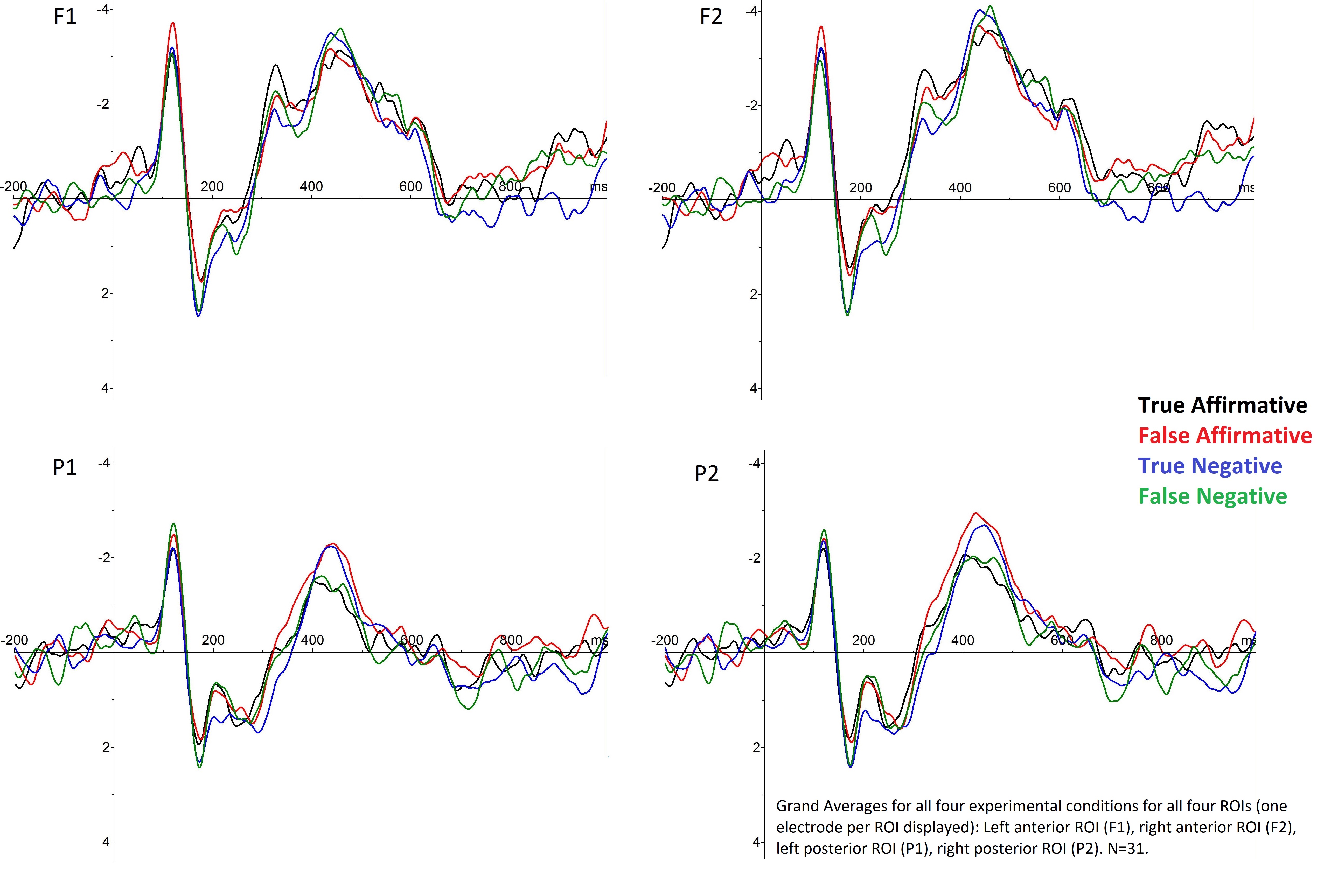

Supplement: Supplementary file 3 [file Image_4.jpg]
